# Molecular Identification of Hookworm Isolates in Humans, Dogs and Soil in a Tribal Area in Tamil Nadu, India

**DOI:** 10.1371/journal.pntd.0004891

**Published:** 2016-08-03

**Authors:** Santosh George, Bruno Levecke, Deepthi Kattula, Vasanthakumar Velusamy, Sheela Roy, Peter Geldhof, Rajiv Sarkar, Gagandeep Kang

**Affiliations:** 1 Department of Gastrointestinal Sciences, Christian Medical College, Vellore, India; 2 Department of Virology, Parasitology and Immunology, Ghent University, Merelbeke, Belgium; Hospital General, MEXICO

## Abstract

**Background:**

Hookworms (*Necator americanus* and *Ancylostoma duodenale*) remain a major public health problem worldwide. Infections with hookworms (e.g., *A*. *caninum*, *A*. *ceylanicum* and *A*. *braziliense*) are also prevalent in dogs, but the role of dogs as a reservoir for zoonotic hookworm infections in humans needs to be further explored.

**Methodology/Principal Findings:**

As part of an open-label community based cluster-randomized trial in a tribal area in Tamil Nadu (India; 2013–2015), a total of 143 isolates of hookworm eggs from human stool were speciated based on a previously described PCR-RFLP methodology. The presence of hookworm DNA was confirmed in 119 of 143 human samples. *N*. *americanus* (100%) was the most prevalent species, followed by *A*. *caninum* (16.8%) and *A*. *duodenale* (8.4%). Because of the high prevalence of *A*. *caninum* in humans, dog samples were also collected to assess the prevalence of *A*. *caninum* in dogs. In 68 out of 77 canine stool samples the presence of hookworms was confirmed using PCR-RFLP. In dogs, both *A*. *caninum* (76.4%) and *A*. *ceylanicum* (27.9%) were identified. Additionally, to determine the contamination of soil with zoonotic hookworm larvae, topsoil was collected from defecating areas. Hookworm DNA was detected in 72 out of 78 soil samples that revealed presence of hookworm-like nematode larvae. In soil, different hookworm species were identified, with animal hookworms being more prevalent (*A*. *ceylanicum*: 60.2%, *A*. *caninum*: 29.4%, *A*. *duodenale*: 16.6%, *N*. *americanus*: 1.4%, *A*. *braziliense*: 1.4%).

**Conclusions/Significance:**

In our study we regularly detected the presence of *A*. *caninum* DNA in the stool of humans. Whether this is the result of infection is currently unknown but it does warrant a closer look at dogs as a potential reservoir.

## Author Summary

Hookworm infections remain a major public health problem in both tropical and subtropical parts of the world. To control the disease burden attributable to hookworms, large-scale deworming programs, in which drugs are administered to schoolchildren regardless of their infection status, are currently being implemented in endemic regions. However, these programs face some difficulties. One of them is the uncertainty about the role of animals in the transmission of hookworm infections. It is commonly believed that human-specific hookworms cause these infections, but there is growing evidence that the role of some animal-specific hookworms as cause of infection in humans should not be underestimated. We determined the different hookworms in humans, dogs and soil (eggs excreted by adult hookworms in stool are non-infectious, and need to develop and hatch on the soil before larvae can transmit disease by penetrating the skin) in a tribal area in India. In this area, the transmission of hookworms between humans and dogs is possible. Our results highlight the presence of DNA from animal-specific hookworms in both soil and human stool. Although these findings suggest that these animals could act as reservoir for zoonotic hookworm infections in humans, they should be interpreted with caution. This is because we lack the evidence to confirm *A*. *caninum* infections in our study population. Other potential reasons for the presence of DNA in stool are contamination of stool with environmental eggs or larvae during sample collection and passive passage in which eggs or larvae are ingested but did result in any infection.

## Introduction

Infections with hookworms (*Necator americanus* and *Ancylostoma duodenale*) remain a major public health problem in several low and middle-income countries [1,2]. It is estimated that ~439 million people are infected, resulting in a global disease burden of ~3.5 million disability adjusted life years (DALYs; 62.3% of the DALYs attributable to soil-transmitted helminths; ~3% of all DALYs attributable to Neglected Tropical diseases (NTDs)) [3]. The major morbidity associated with hookworm infections is caused by intestinal blood loss, iron deficiency anaemia, and protein malnutrition [4], most of which occurs in children and pregnant women [5]. The current strategy to control the morbidity caused by these intestinal worms are embedded in large-scale school-based deworming programs, in which benzimidazole drugs (albendazole and mebendazole) are administered to schoolchildren regardless of their infection status [6,7]. However, it remains unclear whether these school-based deworming programs are the most efficient approach [8].

First, both prevalence and the intensity of hookworm infections increase as a function of age. Although most of the deworming programs target school-aged children, the major contributors of hookworm infection both in terms of prevalence and total egg excretion are adults, who are often not included in deworming programs [6,8]. Second, the eggs excreted in stool are non-infectious, and need to develop and hatch on the soil before larvae can transcutaneously enter the human host and cause disease [9]. Therefore it will be important to supplement deworming programs with improved water, sanitation and hygiene (WASH) to prevent re-infection [10]. Moreover, benzimidazole drugs have a moderate efficacy against hookworm and never reach 100% efficacy [11,12]. Third, it is traditionally assumed that hookworm infections are caused by the human hookworms *N*. *americanus* and *A*. *duodenale*, and hence hookworm infections in humans are solely due to the contamination of soil with human stool [13]. Infections with the hookworms (e.g. *A*. *caninum*, *A*. *ceylanicum*, *A*. *braziliense and Uncinaria stenocephala)* in dogs are also highly prevalent, and depending on the species these hookworms may also cause a variety of clinical symptoms in humans [14]. *A*. *ceylanicum* is the only known species to cause patent infection in humans [15] with symptoms ranging from gastrointestinal discomfort, epigastric pain, flatulence and diarrhoea, whereas the rest are mainly limited to lesions in the skin caused by migrating larvae (cutaneuos larva migrans) [16]. Migration to the intestine has been reported for *A*. *caninum* which may cause severe eosinophilic enteritis [14,17]. Recent studies also indicate that the role of animals as a source of hookworm infections in humans should not be ignored. For example, in a study done in a rural region of Cambodia [18] 64 out of 124 (51.6%) individuals were found to be infected with the animal hookworm *A*. *ceylanicum*, of which the majority were mono-infections (89%) [16]. Similarly, a study done in a tribal region in India [19], found that human hookworm infections (*N*. *americanus* 39/41; 95% and *A*. *duodenale* 6/41; 15%) accounted for majority of the infections, whereas the animal hookworm *A*. *ceylanicum* only accounted for a minority of the infections (2/41; 5%), and hence these findings suggest that the rate of zoonotic transmission might vary across different geographical areas.

Despite these studies, the role of animals as a reservoir for hookworm infections in human remains poorly explored. This lack of understanding of disease transmission among both humans and animals, is largely due to the fact that diagnosis of hookworm infections are based on the microscopic demonstration of eggs in stool, but it is impossible to differentiate animal and human hookworm eggs based on morphology. For this, molecular tools are more appropriate [20]. Second, various studies have identified hookworm species separately across humans [19,21,22], dogs [23,24,25] or soil [26], but to our knowledge there are no studies which have identified hookworm species using molecular techniques within both hosts and environment in the same geographical region. The present study aims at molecular identification of hookworms isolates from humans, dogs and soil from a tribal area in Tamil Nadu, India. The selection of this study area was based on (i) a high prevalence of hookworm infection in humans (38%) [27] and (ii) the presence of factors that facilitates zoonotic hookworm transmission in humans.

## Methods

### Ethics Statement

This study was part of an open-label, community-based cluster randomized trial that was approved by the Institutional Review Board of Christian Medical College, Vellore, India. This trial is registered in the Clinical Trials Registry of India (CTRI/2013/05/003676). A description of the ethical considerations has been described in detail elsewhere (Sarkar *et al*., under review). In short, a written informed consent was obtained from parents/legal guardian for the collection of stool samples from children aged less than 18 years of age and an assent was obtained from 8–17 year old children. Participants older than 18 years of age signed their own informed consent form.

### Study Setting, Sample Selection and Laboratory Procedure

Jawadhu hills are situated in Vellore and Thiruvannamalai district of Tamil Nadu (southern India). It covers an area of 150 km^2^ and a population of approximately 80,000 of which the majority is tribal. The population is organized in 11 ‘panchayat’ (a group of villages under one local administrative council) and 229 villages [28]. The area is known to have red loamy soil [28]. The temperatures of the region ranges between 12°C and 33°C [28]. There is excessive rainfall (>1000 mm) [28], with relative humidity varying from 40 to 85% [28]. The majority of the population is employed in the agricultural sector and lives in close proximity with animals, including dogs and cats. It is important to note that these animals are not confined, and although they belong to one household are found freely roaming through the village. Across the entire area there is a common practice of open-field defecation [27].

The study was part of an open-label, community-based cluster randomized trial conducted between 2013–2015. The aim of the trial was to compare the hookworm re-infection rates for one year in a population that was subjected to varying cycles of deworming using albendazole. Therefore, 15 clusters (villages) were randomized into one of three different treatment arms: (i) single cycle, (ii) two cycles and (iii) four cycles. The timing of deworming in each of the three groups have been described in detail elsewhere (Sarkar *et al*., 2016; under review). In short, in the single cycle, individuals received a single oral dose of albendazole once in the beginning of the study and stool samples were collected 3, 6, 9 and 12 months post-treatment. In the treatment arm of two cycles, individuals received two single oral doses of albendazole. The first dose was given at the start of the trial and second dose after one month. The stool samples were collected 3, 6, 9 and 12 month after the administration of the second dose. In the treatment arm of four cycles, the first two doses of albendazole were given at the start of the trial with one-month interval, and an additional two doses of albendazole were given at 6 months after the 2^nd^ dose. Stool samples were also collected after 3 and 6 months post 2^nd^ dose and 3, 6, 9 and 12 months post 4^th^ dose of albendazole.

In the present study, stool (humans and dogs) and soil were collected from nine clusters included in the trial (3 per treatment arm), including Seramarthur, Jambudee, Alanjanur, Sinthalur, Koothatur, Villichanur, Keel Nadanur, Thimirimarathur and Pudhupattu. In 2013 the total population of the 9 villages was 2,906 habitants (1,492 males and 1,414 females) belonging to 683 families.

Human stool samples were collected as per trial protocol described above. Based on the treatment arm, stool samples were collected at an interval of three months until the end of one year after the last treatment. Field workers visited the house of the study a day before collection was scheduled and handed over a plastic stool container and wooden spatula. Containers were appropriately labelled. The stool samples were collected the following day and stored at the study area at 4°C before being transported to the laboratory using cold containers. In total 2,152 stool samples were collected from 711 individuals from the 9 selected villages. All samples were screened microscopically applying a saline wet mount. Stool in which hookworm eggs were found were subsequently screened using the McMaster egg counting method to estimate the intensity of infection (faecal egg counts (FECs) expressed in number of eggs per gram of stool (EPG)). Finally, one stool sample per infected subject was withheld for molecular analysis, and stored at -70°C. If hookworm eggs were found in multiple samples from the same individual, the sample with the highest FEC was selected. A total of 146 out of the 711 individuals were found to be excreting eggs in at least one time point. The median (25^th^ quantile (Q_25_) - 75^th^ quantile (Q_75_)) FEC among the infected individuals was 550 EPG (200–1,000). The number of infected humans and the corresponding median FEC across the different villages are summarized in Table 1.

**Table 1:** Molecular characterization of hookworm across 9 villages in human stool collected from Jawadhu hills.

Village	N	Microscopy positive	Median FEC	PCR positive	Molecular characterization of hookworm		
			(Q_25_—Q_75_)		*N*. *americanus*	*A*. *duodenale*	*A*. *caninum*
Alanjanur	59	7	250 (150–1650)	6	6	0	6
Jambudee	75	26	500 (200–950)	20	20	1	2
Keel Nadanur	74	14	1,300 (500–1700)	13	13	0	5
Kootathur	107	22	550 (250–750)	21	21	0	4
Pudhupattu	120	11	300 (150–650)	8	8	0	1
Seramarathur	63	23	1,100 (500–3450)	23	23	7	1
Sinthalur	70	8	200 (125–450)	5	5	0	1
Thimirimarathur	55	11	200 (100–450)	6	6	1	0
Villichanur	88	21	650 (300–900)	17	17	1	0
**Total**	**711**	**143 (20.1%)**	**550 (200–1000)**	**119 (83.2%)**	**119 (100%)**	**10 (8.4%)**	**20 (16.8%)**

FEC—Fecal egg count expressed as eggs per gram of stool

Q_25_:25^th^ quantile; Q_75:_ 75^th^ quantile of an ordered range of data.

Field workers identified 10 houses per village based on structured questionnaire that had previously claimed dog ownership, and volunteered to help collect stool samples. As the dogs in these villages were not usually confined, the owners chained their dogs for a day and stool sample was collected into the plastic stool container using a spatula after the dog defecated. In each village, a single stool sample from 10 dogs was collected (n = 90). The stool samples were collected and stored at 4°C at the site of collection. The samples were transported to the laboratory in cold containers. As with human stool samples, dog stool were first screened with saline wet mount, after which stool of infected animals were re-examined using the McMaster egg counting method. All samples containing hookworm eggs were withheld for molecular analysis, and stored at -70°C. In total, 77 out of 90 dogs excreted hookworm eggs in stool. The median (Q_25_—Q_75_) FEC across infected dogs was 350 EPG (100–650). The number of infected dogs and the corresponding median FEC across the different villages are summarized in Table 2.

**Table 2:** Molecular characterization of hookworm across 9 villages in dog stool collected from Jawadhu hills.

Village	N	Microscopy positive	Median FEC	PCR positive	Molecular characterization of hookworm	
			(Q_25_—Q_75_)			
					*A*. *caninum*	*A*. *ceylanicum*
Alanjanur	10	10	875 (450–1350)	9	5	6
Jambudee	10	7	100 (50–850)	5	2	3
Keel Nadanur	10	8	350 (75–475)	8	8	0
Kootathur	10	8	150 (50–200)	8	8	1
Pudhupattu	10	10	300 (150–2200)	10	9	1
Seramarathur	10	8	525 (250–1875)	7	5	2
Sinthalur	10	8	450 (300–775)	7	6	1
Thimirimarathur	10	10	500 (250–550)	7	3	4
Villichanur	10	8	325 (175–350)	7	6	1
**Total**	**90**	**77 (85.5%)**	**350 (100–650)**	**68 (88.3%)**	**52 (76.4%)**	**19 (27.9%)**

FEC: Fecal egg count expressed as eggs per gram of stool; Q_25_: 25^th^ quantile; Q_75_: 75^th^ quantile

Soil samples were collected from common open defecation areas for each of these villages. Field workers opportunistically collected soil samples from hot spots of the defecation site chosen for the study. The soil samples that were collected were found to be loamy and wet. The collection of soil samples was in conjunction with human stool samples. Hookworm larvae are known to be present in the top soil early in the morning [29,30] and therefore sample collection was done between 8 a.m. and 10 a.m. Depending on the number of open defecation areas in a village and the number of cycles of deworming, 20 to 40 soil samples were collected per village. Approximately 250–300 grams of topsoil was collected and transported in plastic bags at room temperature to the laboratory on the same day. All samples were screened for the presence of hookworm-like nematode larvae applying a modified saline wet mount. In total, 271 samples were collected from 22 open defecation sites. In 78 samples out of 271 samples hookworm-like nematode larvae were identified. The total number of soil samples collected and the number of soil samples containing hookworm-like nematode larvae across the different villages are summarized in Table 3. All samples were stored at 4°C until the molecular identification of the larvae.

**Table 3:** Molecular characterization of hookworm across 9 villages in soil samples collected from Jawadhu hills.

Village	N	Microscopy positive	PCR positive	Molecular characterization of hookworm				
				*A*. *caninum*	*A*. *ceylanicum*	*A*. *duodenale*	*A*. *braziliense*	*N*. *americanus*
Alanjanur	40	11	10	5	4	1	0	0
Jambudee	30	11	10	2	7	1	0	0
Keel Nadanur	30	5	5	1	4	0	0	0
Kootathur	40	21	20	6	12	2	1	0
Pudhupattu	31	5	4	1	3	0	0	0
Seramarathur	20	5	4	1	2	1	0	0
Sinthalur	20	2	2	1	0	1	0	0
Thimirimarathur	40	10	9	5	3	2	0	1
Villichanur	20	8	8	1	6	1	0	0
**Total**	**271**	**78 (28.8%)**	**72 (92.3%)**	**20 (27.7%)**	**41 (56.9%)**	**8 (11.1%)**	**1 (1.4%)**	**1 (1.4%)**

#### Microscopic examination

The saline wet mount was performed on stool as described in laboratory manual by WHO [31]. For soil samples, two grams of soil sample was suspended in 10 ml buffered saline (0.85% NaCl). The suspension was subsequently filtered twice using a tea strainer to withhold any large debris. The obtained filtrate was transferred into a 15 ml falcon tube, and saline was added up to a volume of 10 ml. The suspension was centrifugation at 3,150 g for 10 minutes. The supernatant was discarded and a saline wet mount was performed.

McMaster egg counting method was performed as described by Levecke and colleagues, 2011 [32]. In short, two grams of stool were suspended in 30 ml of saturated NaCl solution (specific gravity ∼1.2). The fecal suspension was run through filter (250 μm) three times to remove any large debris. Then, 500 μl of the remaining suspension was added to each of the two chambers of a McMaster slide (http://www.mcmaster.co.za). Both chambers were examined under a light microscope using a 100x magnification and the FEC, expressed as EPG for hookworms, were obtained by multiplying the total number of hookworm eggs by 50. A visual tutorial on how to perform a McMaster can be found at https://www.youtube.com/watch?v=bwIFyZ7NrFw.

#### Molecular identification of hookworm isolates in stool samples

DNA was extracted from stool using the Qiagen stool DNA-mini kit (Qiagen, Hilden, Germany). One gram of stool sample was mixed with 1 ml of lysis buffer and thoroughly mixed using a vortex for 10 minutes. Subsequently, 0.1 gram of 425–600 μm acid-washed glass beads was added to the suspension and was continuously beaded at variable shaking speeds of 2,000–3,450 strokes/min for 9 minutes. To further enhance the recovery of DNA, the samples were then subjected to 5 freeze-thaw cycles; first putting the samples in water bath of 95°C for 5 minutes followed by a freeze step in liquid nitrogen for 2 minutes. With the exception of the elution step, which was repeated twice, the remaining of the DNA extraction was performed according to the manufacturer’s protocol.

#### Molecular identification of hookworm isolates in soil samples

Prior to DNA extraction, larvae were isolated from the soil. To this end, 20 grams of the soil samples was suspended in 10 ml of distilled water. The choice of the amount of soil was based on larval recovery rates across 2, 4, 5, 10 and 20 grams of soil. Subsequently, the sample was filtered twice through a tea strainer to retain any larger debris. The obtained filtrate was subjected once more to 2 consecutive filtration steps, using a sieve filter of pore size 0.4 mm and 0.2 mm respectively. The remaining material was then scrapped off the 0.2 mm sieve filter where the larvae retained and transferred into a 15 ml falcon tube, and 3 ml of MgSO_4_ solution (specific gravity = 1.2). The suspension was centrifuged at 2,000 g for 2 minutes, the supernatant was transferred into a new 15 ml falcon tube, and 3 ml of distilled water was added. Finally, the end solution was concentrated to 1 ml, which was then processed using the Qiagen Blood and Tissue kit (Qiagen, Hilden, Germany).

#### Identification of hookworm species

The ITS 1,2 and 5.8s region of the hookworm genome was amplified using a semi-nested PCR protocol described by George and colleagues, 2015 [19]. The first-round PCR resulted in a product of 597 bp (*N*. *americanus*) and in a product of 449 bp (*Ancylostoma* spp. and *U*. *stenocephala*), while the second PCR resulted in a product of 552 bp (*N*. *americanus*), 404–408 bp (*Ancylostoma* spp. and *U*. *stenocephala*). Both a negative (water) and positive (hookworm DNA) control was included in each run. The amplification reactions and conditions have been described in detail elsewhere [19]. The amplified product was detected using 1.5% agarose gel electrophoresis using ethidium bromide. To determine the species of *Ancylostoma*, the second-round PCR products were subsequently digested using the restriction enzymes as mentioned by George *et al*., 2015 [19]. To differentiate between *A*. *braziliense*, *A*. *ceylanicum*, *A*. *caninum and A*. *duodenale*, DNA from these species were subjected to two different restriction enzymes (MvaI and Psp1406I) at 37°C for 13 hours. MvaI digest *A*. *braziliense* into three fragments of 64, 122 and 222 bp using and *A*. *ceylanicum* into two fragments of 255 and 149 bp; but is not able to digest *A*. *duodenale*, *A*. *caninum* and *U*. *stenocephala*. Psp1406I digests both *A*. *braziliense* and *A*. *duodenale* into two fragments (*A*. *braziliense*: 259 and 149 bp; *A*. *duodenale*: 255 and 149 bp), but not *A*. *caninum*. The restricted products were detected using 2% agarose gel electrophoresis and ethidium bromide. Based on previous findings, the likelihood of observing the canine *U*. *stenocephala* and the feline hookworm *A*. *tubaeforme* was expected to be low in our study setting [33], and hence these hookworm species were not prioritized while selecting RFLP enzymes. Our analysis on the reference sequence from GenBank for both *A*. *tubaeforme* and *U*. *stenocepahala* revealed that both MvaI and Psp1406I do not have restriction sites in their genome. To confirm the specificity of the PCR-RFLP method and to exclude any *U*. *stenocephala* and *A*. *tubaeforme* infections, DNA sequencing was performed on a subset of the isolates. DNA sequencing was done using a dye terminator cycle sequencing kit and a four-capillary array genetic analyser (Applied Biosystems 3130) directly from the purified amplicons, which were sequenced in both directions using the same oligonucleotide primers used by George *et al*., 2015 [19]. In the presence of mixed infections, the amplified products were run on 1.5% agarose gel and the desired product cut from the gel and purified using fast bind-wash-elute method using QIAquick gel extraction kit. Sequences were aligned by pairwise alignment using the ClustalW method with MegAlign DNASTAR software. The pairwise alignment was done to draw inference on the relationship between hookworms from humans, dogs and soil. A bootstrap consensus tree inferred from 100 replicates was used to generate the tree. For each of the different hookworm species reference samples were included (*A*. *braziliense*: GenBank accession no DQ359149; *A*. *caninum*: GenBank accession no DQ438070; *A*. *ceylanicum*: GenBank accession no DQ381541; *A*. *duodenale*: GenBank accession no EU344797 and *N*. *americanus*: GenBank accession no AB793527).

#### Analytic sensitivity of detecting *N*. *americanus* L_3_-larvae in soil

In order to verify the analytic limit of detecting *N*. *americanus* L_3_-larvae recovered from soil, a spiking experiment was set up. To this end, a series of known number of *N*. *americanus* L_3_-larvae were added to a fixed aliquot of sterile soil. In this experiment 70, 140, 280 and 700 larvae were added to 20 gram of soil, resulting in a concentration of 3.5, 7, 14 and 35 larvae per gram of soil. Each of the number of larvae was added to 5 different aliquots of soil, resulting in 20 seeded aliquots of soil. These seeded aliquots were processed as described above in the section *Molecular identification of hookworm isolates*. Prior to DNA extraction, the number of larvae isolated from 20 gram of soil was determined by microscopically screening 4x 10 μl of the final 1 ml elute.

The L_3_ larvae were obtained by applying the Harada-Mori method to human stool submitted to the laboratory for routine microscopic examination. The larvae were kept at a concentration of 14,000 larvae / ml, and hence 70, 140, 280 and 700 larvae were represented by a larval stock volume 5 μl, 10 μl, 20 μl and 50 μl, respectively. To fix the total volume added to the soil, sterile water was added up to a volume of 1,000 μl for all experiments. One kilo of soil, which was negative on the saline wet mount, was dry heat sterilized (160°C for 2 hours). The sample was subsequently processed as described in *Molecular Identification of Hookworm Isolates* and found to be negative for *N*. *americanus*-DNA.

## Results

### Molecular Characterization of Hookworm in Humans, Dogs and Soil

From the 711 individuals that participated in the study, 146 individuals were found to be infected with hookworm using saline wet mount microscopy. Out of the 146 infected individuals, 143 individuals provided adequate quantity of stool samples to perform molecular characterization, and hookworm-DNA was detected in 119 individuals (83.2%). All had *N*. *americanus*, while *A*. *caninum* was found in 20 individuals and *A*. *duodenale* in 10 individuals. The distribution of the different hookworm species in human stool across the 9 villages is summarized in Table 1. Based on a structured questionnaire, the odds of being infected with *A*. *caninum* when claiming dog ownership (11/299) was 1.71 (95% confidence interval = 0.69–4.18) times higher when no dog ownership was claimed (9/412), but this was not statistically significant.

On account of high prevalence of *A*. *caninum* DNA in human stool samples, dog stool samples were collected from the 9 villages. A total of 90 dog stool samples were collected, of which hookworm was detected in 77 samples using saline wet mount microscopy. Hookworm was detected in 68 of the 77 dog samples (88.3%) selected for molecular characterization. *A*. *caninum* was the predominant species, being found in 52 dogs. *A*. *ceylanicum* was found in 19 (27.9%) dogs. Among them three dogs had mixed *A*. *caninum* and *A*. *ceylanicum* infections. The distribution of the different hookworm species across the 9 villages is summarized in Table 2.

To assess the role of soil as a source of zoonotic hookworm infection, a total of 271 soil samples were collected from defecating areas across 9 villages. Hookworm-like nematode larvae were found in 78 out of the 271 samples that were collected. Of the 78 soil samples identified positive for hookworm-like nematode larvae, 72 (92.3%) were found positive by PCR for hookworms. Molecular characterization of these 78 soil samples revealed the presence of a variety of hookworm species, including *A*. *caninum*, *A*. *ceylanicum*, *A*. *duodenale*, *A*. *brazilense* and *N*. *americanus*. The majority of these soil samples were contaminated with *A*. *ceylanicum* (n = 41; 56.9%), followed by *A*. *caninum* (n = 20; 27.7%) and *A*. *braziliense* (n = 1; 1.4%). The human hookworms were only found in the minority of the samples (*A*. *duodenale*: n = 8; 11.1%; *N*. *americanus*: n = 1; 1.4%). The distribution of the hookworm species across the different villages is summarized in Table 3.

### Sequence Analysis

**Fig 1:**
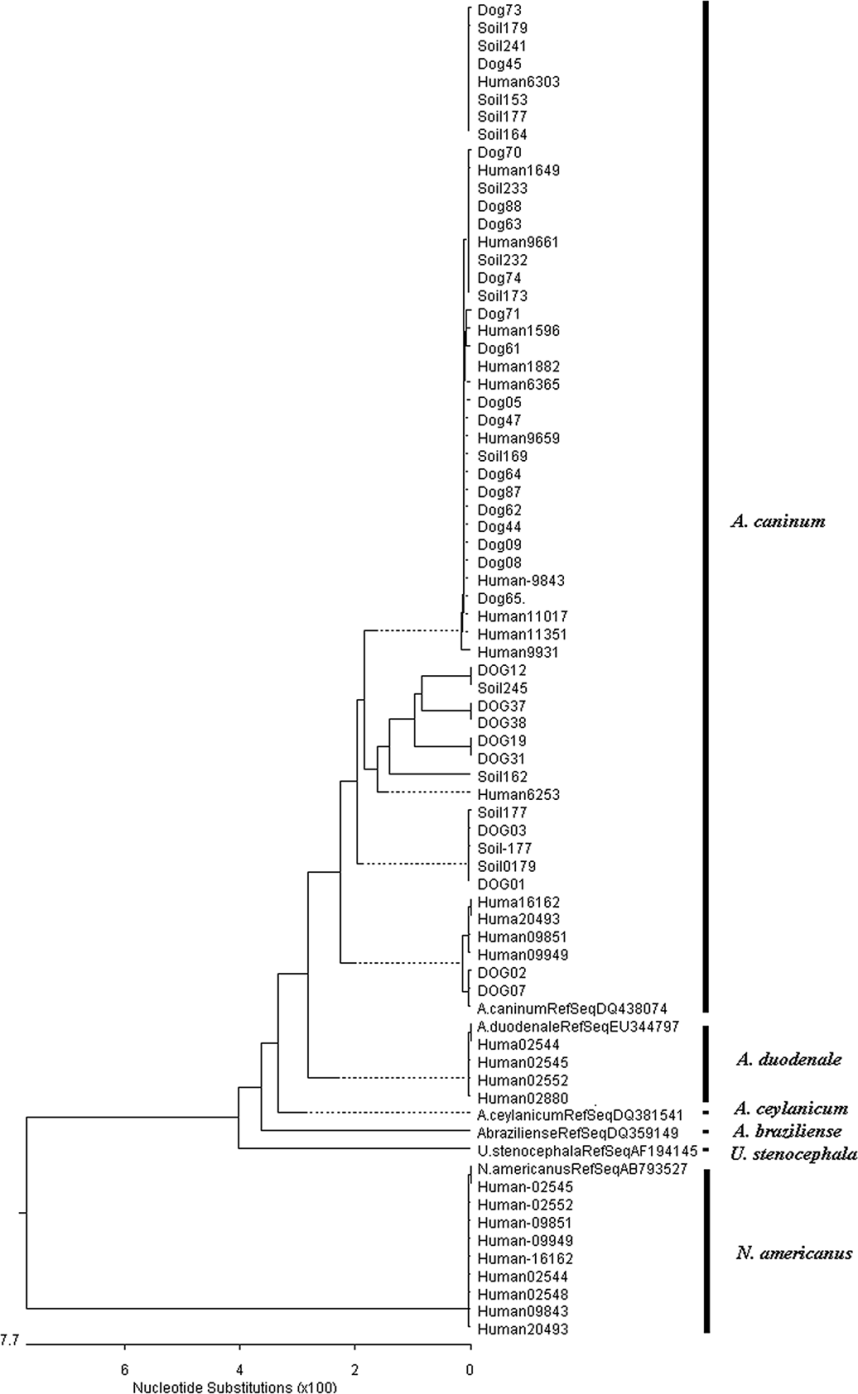
Phylogenetic tree constructed using Pairwise Alignment to draw inferences on relationship between different hookworm species. Sequences were aligned by pairwise alignment using the ClustalW method with MegAlign DNASTAR software. The pairwise alignment was done to draw inference on the relationship between hookworms from humans, dogs and soil. A bootstrap consensus tree inferred from 100 replicates was used to generate the tree.

In total 69 hookworm isolates from different sources (29 human, 26 Dog and 14 soil) were sequenced in the present study. The phylogenetic tree is provided in Fig 1, and highlights that each species identified by the PCR-RFLP cluster nicely together with their respective reference sequences. The human-derived *A*. *caninum* sequences (Study ID nos. Human20493, Human16162, Human11351, Human11017, Human9949, Human9931, Human9851, Human9843, Human9661, Human9659, Human6365, Human6303, Human6253, Human1882, Human1649 and Human1596) clustered with the *A*. *caninum* sequences from dogs and soil, forming a cluster with *A*. *caninum* reference sequences (GenBank accession no. DQ438070). The human-derived sequences of *A*. *duodenale* (Study ID nos. Human2545, Human2880, Human2552 and Human2544) centred within a clade with the reference *A*. *duodenale* sequence (GenBank accession no. EU344797). The *N*. *americanus* sequences from humans clustered as a separate larger cluster. All 69 sequences were submitted to GenBank and assigned the accession numbers from KU996361 to KU996390, and from KX155777 to KX155815

### Analytic Sensitivity of Detecting *N*. *Americanus* L_3_-Larvae in Soil

Due to low detection of *N*. *americanus* larvae in the soil samples but the high prevalence of hookworm (*N*. *americanus*) in humans, an experiment was carried out to assess the analytic sensitivity of detecting *N*. *americanus* larvae in soil samples using the standardized assay as described in material and methods section:. For the various concentrations (70, 140, 280 and 700 larvae) of the larvae in the stock that was used to spike, there was a large variation in mean recovery rate (%) of the *N*. *americanus* L_3_-larvae, ranging from 51.1% when 70 larvae were added to 92.9% when 700 larvae were added. The results of the larvae recovery rate are presented in Table 4. The larvae that were isolated from these experiments were characterized using PCR and were confirmed to be *N*. *americanus*.

**Table 4:** The recovery rates (%) with different larval concentrations in five batches of soil aliquots.

N	Mean (SD*)	Recovery rate (%)
Larvae added to the soil	Recovered larvae	
70	40 (13.7)	57.1
140	125 (0)	89.3
280	255 (11.2)	91.1
700	650 (30.6)	92.9

*SD: standard deviation

## Discussion

There has been a worldwide upscale of drug donations to control the morbidity caused by hookworms, and to even attempts to eliminate these worms in confined geographical areas [6]. It is traditionally assumed that infections in humans are solely due to the human hookworms (*N*. *americanus* and *A*. *duodenale*) [6,34], hence ignoring the possible role of animals as a reservoir for hookworm infections. This study determined the hookworm species in humans, dogs and soil from a tribal area in Tamil Nadu, India. The findings from our present work confirm that *N*. *americanus* are responsible for the majority of the hookworm infections in humans in these tribal communities [19]. In addition, we also found an unexpectedly high prevalence of animal hookworm DNA in humans, while our previous study [19] in Jawadhu hills had a low prevalence of animal hookworms (5%). These differences in occurrence might be explained by variation in prevalence across villages. Both studies covered different villages, and as illustrated in Table 1, there was a large variation in animal hookworm infections in humans across villages (*A*. *caninum* was found in 6 out 6 cases in Alanjanur, but was absent in Thimirimarathur and Villichanur). There was no significant evidence of an increased risk of hookworm infections with dog ownership, which is in contrast with the studies reported by Traub *et al*., and Ugbomoiko US *et al*., [24,35], who did observe a significant increased risk. The lack of this evidence maybe due to the fact that animals are not confined, but are found freely roaming through the village. As a consequence of this they are able to randomly defecate within the village, which subsequently will increase the likelihood of infecting other habitants beyond their owner.

In the present study a large proportion of human stools were found to contain *A*. *caninum* DNA. These observations can be explained by (i) *A*. *caninum* infections, (ii) passive passage of *A*. *caninum* eggs or larvae that are accidently ingested, but do not result in any infection and (iii) contamination of stool during sample collection with environmental *A*. *caninum* eggs or larvae. Although it is unlikely that passive passage explains the high proportion of stool samples containing *A*. *caninum* DNA, we have no conclusive evidence for any of the remaining potential causes either. Traditionally it is assumed that parasite DNA in stool is due the presence of eggs shed by adult worms. However, up to today there is no evidence yet that egg-laying adult *A*. *caninum* worms can develop in humans [14,36,37], and hence one would not expect any amplification of DNA from *A*. *caninum* extracted eggs in human stool. In our study we were not able to provide evidence for the presence of *A*. *caninum* eggs in stool, as the human hookworm *N*. *americanus* was also detected in all cases of *A*. *caninum*. As a consequence of this, it is possible that the eggs in stool were shed by adult *N*. *americanus* worms only. A single-egg based speciation would have been ideal. Another potential source of parasite DNA is DNA that is directly released by immature or mature non-egg producing worms. To demonstrate the presence of both immature and non-egg producing mature *A*. *caninum* worms expulsion studies are required [36]. In these studies stool is collected over consecutive days following treatment to recover worms, which are then individually speciated. Both a single-egg based speciation and an expulsion study were out of scope of the present study. Another aspect that needs to be considered during the interpretation of our findings is the way the human samples were collected. Although all the study participants were informed about the importance of the study and need to collect fecal samples devoid of any soil, stool samples could have been contaminated with soil during collection because people in the study area defecate in the open and samples might have been scooped from the ground which could result in the presence of *A*. *caninum* DNA in human stool. In either case, these results emphasize the need for additional epidemiological surveys across various geographical settings to further explore the role of animals as a reservoir for zoonotic transmission. It is important to note that this does not only apply for hookworms, but is also of concern for other soil-transmitted helminths (*Trichuris trichiura* and *Ascaris lumbricoides*). This is because dogs are known to harbor *Trichuris* spp. (*T*. *vulpis*), which, similarly to canine hookworm species, is known to infect humans causing symptoms ranging from an asymptomatic infection to diarrhea or even dysentery [38]. *T*. *vulpis* has also been reported as a causative agent of visceral larva migrans [39,40,41].

In the dog stool samples, both known zoonotic hookworm (*A*. *caninum* and *A*. *ceylanicum*) species were found. Although *A*. *ceylanicum* is the only canine hookworm species that is known to cause patent infections in humans [42], our present study did not identify any human *A*. *ceylanicum* infections, in spite of detecting it in both soil and canine stool samples. In contrast, a study from a rural village in Cambodia reported more than half of the hookworm infected individuals to be positive for *A*. *ceylanicum* (51.6%) [18] A possible explanation for the presence of *A*. *ceylanicum* in dogs and soil, but not in humans, is the existence of two sub species (haplotypes) of *A*. *ceylanicum*, one with an animal origin and one with a human origin [42]. To differentiate these subspecies the cytochrome c oxidase (COX) subunit 1 gene of the hookworm is recommended [42]. In addition, the applied PCR-RFLP method may lack some sensitivity; this is in particular for mixed infections. As previously illustrated for other gastro-intestinal parasites (e.g. *Giardia*; Geurden *et al*., 2008 and Levecke *et al*., 2009), genus specific PCRs will preferentially amplify the most abundant species, and hence presence of the least abundant species may be underestimated [43,44]. This could be one possible explanation for missing out zoonotic *A*. *ceylanicum* infections in humans. For the soil samples that were collected from defecating areas, it was interesting to observe variety of different species of hookworm larvae (*A*. *braziliense*, *A*. *caninum*, *A*. *ceylanicum*, *A*. *duodenale* and *N*. *americanus*). This can be attributed to open defecation practised in the study area and indiscriminate defecation by freely roaming stray dogs.

There are a few limitations to our present study. First, *N*. *americanus* was rarely found in soil samples, whereas this hookworm species was found in all human stool samples. For this reason, we determined the analytical sensitivity of the isolation procedure using *N*. *americanus* L_3_-larvae to confirm efficiency of the assay to isolate and identify the species. The results of this seeding experiment suggest that the procedure was able to detect 3.5 larvae per gram of soil, and hence ruling out false negative test results due to loss of larvae. Another potential cause of absence of *N*. *americanus* could be inappropriate storage of the soil samples prior to the molecular analysis. In this study, the soil samples were first processed for hookworm-like larvae using modified saline wet mount microscopy, subsequently they were stored at 4°C (up to 14 months) until further processed for molecular identification. Unlike *Ancylostoma* spp., *N*. *americanus* stored in cold temperature do not survive long [29]. It is therefore important to mention that the only case of *N*. *americanus* was observed in one out of four samples containing hookworm-like larvae that were processed almost immediately after collection for molecular speciation.

Second, for the collection of soil samples, the samples were collected from area around the site of defecation/presence of stool (human and dog), which makes the selection biased, and hence it increased the probability of finding hookworm larvae.

In conclusion, in our study we regularly detected the presence of *A*. *caninum* DNA in the stool of humans. Whether this is the result of an infection is currently unknown but it does warrant a closer look at dogs as a potential reservoir. Nevertheless, there is a need for additional epidemiological surveys across different geographical settings to further unravel the role of animals as a reservoir for zoonotic transmission, and ultimately inform the health policy makers to adapt or improve measures to control soil-transmitted helminths as a public health problem.

## Supporting Information

S1 Appendix Determination of the required quantity of soil for effective isolation of larvae. (DOCX)

S1 Checklist STROBE Checklist: STROBE Statement—checklist of items that should be included in reports of observational studies. (DOC)
