# Supplementary material for: Molecular Identification of Hookworm Isolates in Humans, Dogs and Soil in a Tribal Area in Tamil Nadu, India
Source: PLoS Negl Trop Dis. 2016 Aug 3;10(8):e0004891. doi: 10.1371/journal.pntd.0004891 (PMC4972381; doi:10.1371/journal.pntd.0004891)
Supplement: S1 Appendix — (DOCX) [file pntd.0004891.s001.docx]

**Supporting information 1: Determination of the required quantity of soil for effective isolation of larvae**

In order to determine the required quantity of soil for effective isolation of larvae, we conducted the following experiment. We assessed the isolation of larvae from different quantities (2, 4, 5, 10 and 20 grams) of 5 soil samples known to contain larvae. The larvae were isolated as described in the manuscript. Subsequently, DNA was extracted and a nested PCR was performed on each of these isolates to detect hookworm DNA. The results of this experiment are summarized in Table below, indicating that at least 20 grams of soil is required to successfully isolate and amplify hookworm DNA.

Table 1 The presence of hookworm larvae and DNA in isolates obtained from different quantities of soil.

| **Sample ID** | **Amount of soil (grams)** | **Presence of larvae in isolate** | **Amplification of hookworm DNA** |
| --- | --- | --- | --- |
| 1 | 2 | No | No |
|  | 4 | No | No |
|  | 5 | No | No |
|  | 10 | No | No |
|  | 20 | Yes | Yes |
| 2 | 2 | No | No |
|  | 4 | No | No |
|  | 5 | No | No |
|  | 10 | No | No |
|  | 20 | Yes | Yes |
| 3 | 2 | No | No |
|  | 4 | No | No |
|  | 5 | No | No |
|  | 10 | No | No |
|  | 20 | Yes | Yes |
| 4 | 2 | No | No |
|  | 4 | No | No |
|  | 5 | No | No |
|  | 10 | No | No |
|  | 20 | Yes | Yes |
| 5 | 2 | No | No |
|  | 4 | No | No |
|  | 5 | No | No |
|  | 10 | No | No |
|  | 20 | Yes | Yes |
